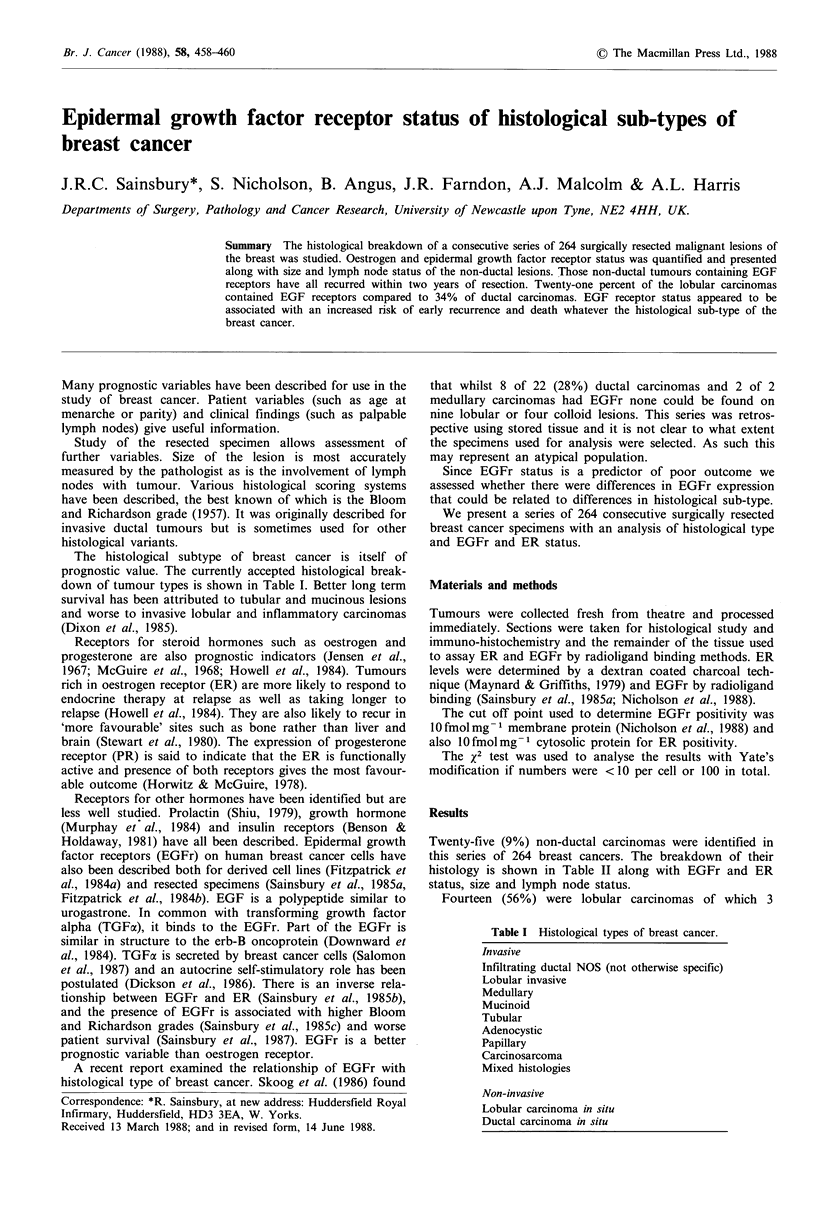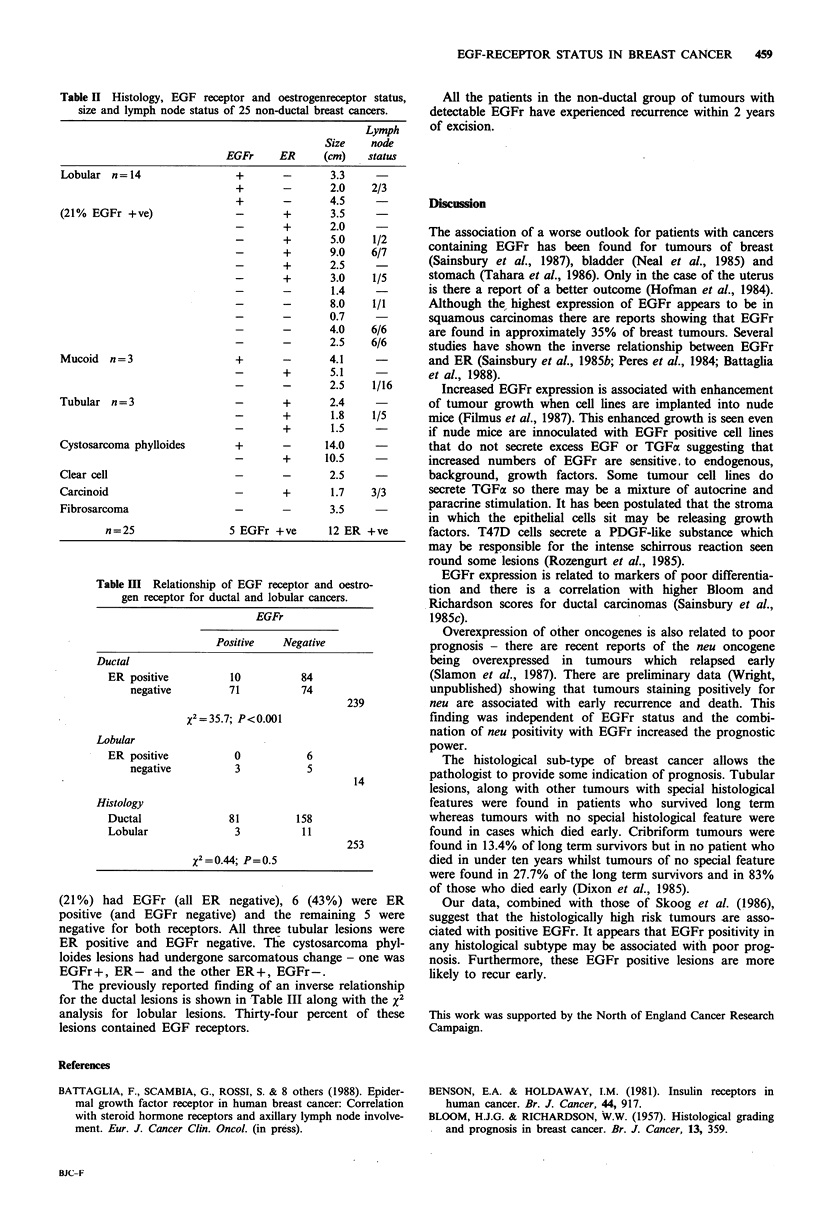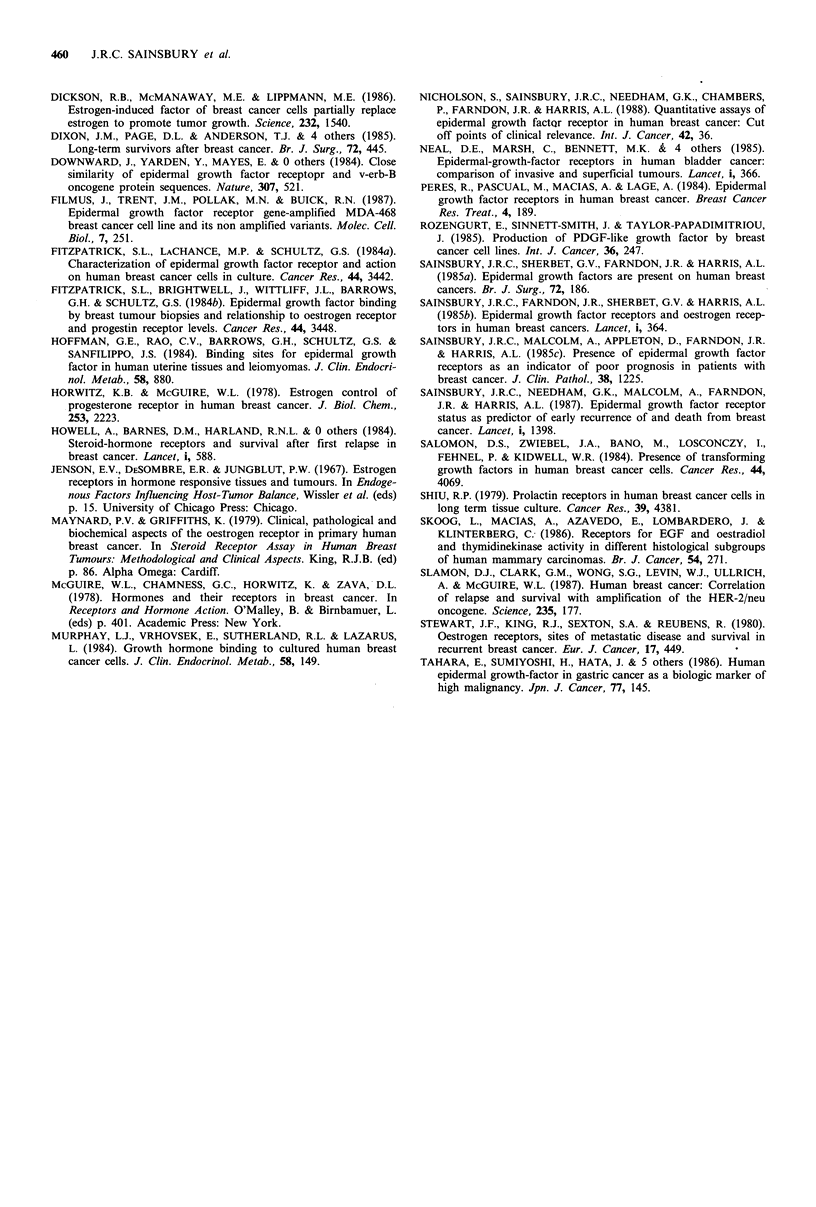# Epidermal growth factor receptor status of histological sub-types of breast cancer.

**DOI:** 10.1038/bjc.1988.240

**Published:** 1988-10

**Authors:** J. R. Sainsbury, S. Nicholson, B. Angus, J. R. Farndon, A. J. Malcolm, A. L. Harris

**Affiliations:** Department of Surgery, University of Newcastle upon Tyne, UK.

## Abstract

The histological breakdown of a consecutive series of 264 surgically resected malignant lesions of the breast was studied. Oestrogen and epidermal growth factor receptor status was quantified and presented along with size and lymph node status of the non-ductal lesions. Those non-ductal tumours containing EGF receptors have all recurred within two years of resection. Twenty-one percent of the lobular carcinomas contained EGF receptors compared to 34% of ductal carcinomas. EGF receptor status appeared to be associated with an increased risk of early recurrence and death whatever the histological sub-type of the breast cancer.


© The Macmillan Press Ltd., 1988

### Epidermal growth factor receptor status of histological sub-types of breast cancer

J.R.C. Sainsbury*, S. Nicholson, B. Angus, J.R. Farndon, A.J. Malcolm & A.L. Harris

*Departments of Surgery, Pathology and Cancer Research, University of Newcastle upon Tyne, NE2 4HH, UK.*

**Summary** The histological breakdown of a consecutive series of 264 surgically resected malignant lesions of the breast was studied. Oestrogen and epidermal growth factor receptor status was quantified and presented along with size and lymph node status of the non-ductal lesions. Those non-ductal tumours containing EGF receptors have all recurred within two years of resection. Twenty-one percent of the lobular carcinomas contained EGF receptors compared to 34% of ductal carcinomas. EGF receptor status appeared to be associated with an increased risk of early recurrence and death whatever the histological sub-type of the breast cancer.

Many prognostic variables have been described for use in the study of breast cancer. Patient variables (such as age at menarche or parity) and clinical findings (such as palpable lymph nodes) give useful information.

Study of the resected specimen allows assessment of further variables. Size of the lesion is most accurately measured by the pathologist as is the involvement of lymph nodes with tumour. Various histological scoring systems have been described, the best known of which is the Bloom and Richardson grade (1957). It was originally described for invasive ductal tumours but is sometimes used for other histological variants.

The histological subtype of breast cancer is itself of prognostic value. The currently accepted histological breakdown of tumour types is shown in Table I. Better long term survival has been attributed to tubular and mucinous lesions and worse to invasive lobular and inflammatory carcinomas (Dixon *et al.*, 1985).

Receptors for steroid hormones such as oestrogen and progesterone are also prognostic indicators (Jensen *et al.*, 1967; McGuire *et al.*, 1968; Howell *et al.*, 1984). Tumours rich in oestrogen receptor (ER) are more likely to respond to endocrine therapy at relapse as well as taking longer to relapse (Howell *et al.*, 1984). They are also likely to recur in 'more favourable' sites such as bone rather than liver and brain (Stewart *et al.*, 1980). The expression of progesterone receptor (PR) is said to indicate that the ER is functionally active and presence of both receptors gives the most favourable outcome (Horwitz & McGuire, 1978).

Receptors for other hormones have been identified but are less well studied. Prolactin (Shiu, 1979), growth hormone (Murphay *et al.*, 1984) and insulin receptors (Benson & Holdaway, 1981) have all been described. Epidermal growth factor receptors (EGFr) on human breast cancer cells have also been described both for derived cell lines (Fitzpatrick *et al.*, 1984*a*) and resected specimens (Sainsbury *et al.*, 1985*a*, Fitzpatrick *et al.*, 1984*b*). EGF is a polypeptide similar to urogastrone. In common with transforming growth factor alpha (TGFα), it binds to the EGFr. Part of the EGFr is similar in structure to the erb-B oncoprotein (Downward *et al.*, 1984). TGFα is secreted by breast cancer cells (Salomon *et al.*, 1987) and an autocrine self-stimulatory role has been postulated (Dickson *et al.*, 1986). There is an inverse relationship between EGFr and ER (Sainsbury *et al.*, 1985*b*), and the presence of EGFr is associated with higher Bloom and Richardson grades (Sainsbury *et al.*, 1985*c*) and worse patient survival (Sainsbury *et al.*, 1987). EGFr is a better prognostic variable than oestrogen receptor.

A recent report examined the relationship of EGFr with histological type of breast cancer. Skoog *et al.* (1986) found that whilst 8 of 22 (28%) ductal carcinomas and 2 of 2 medullary carcinomas had EGFr none could be found on nine lobular or four colloid lesions. This series was retrospective using stored tissue and it is not clear to what extent the specimens used for analysis were selected. As such this may represent an atypical population.

Since EGFr status is a predictor of poor outcome we assessed whether there were differences in EGFr expression that could be related to differences in histological sub-type.

We present a series of 264 consecutive surgically resected breast cancer specimens with an analysis of histological type and EGFr and ER status.

### Materials and methods

Tumours were collected fresh from theatre and processed immediately. Sections were taken for histological study and immuno-histochemistry and the remainder of the tissue used to assay ER and EGFr by radioligand binding methods. ER levels were determined by a dextran coated charcoal technique (Maynard & Griffiths, 1979) and EGFr by radioligand binding (Sainsbury *et al.*, 1985*a*; Nicholson *et al.*, 1988).

The cut off point used to determine EGFr positivity was $10\,\text{fmol}\,\text{mg}^{-1}$ membrane protein (Nicholson *et al.*, 1988) and also $10\,\text{fmol}\,\text{mg}^{-1}$ cytosolic protein for ER positivity.

The $\chi^2$ test was used to analyse the results with Yate's modification if numbers were $<10$ per cell or 100 in total.

### Results

Twenty-five (9%) non-ductal carcinomas were identified in this series of 264 breast cancers. The breakdown of their histology is shown in Table II along with EGFr and ER status, size and lymph node status.

Fourteen (56%) were lobular carcinomas of which 3

**Table I** Histological types of breast cancer.

| |
|---|
| *Invasive* |
| Infiltrating ductal NOS (not otherwise specific) |
| Lobular invasive |
| Medullary |
| Mucinoid |
| Tubular |
| Adenocystic |
| Papillary |
| Carcinosarcoma |
| Mixed histologies |
| *Non-invasive* |
| Lobular carcinoma *in situ* |
| Ductal carcinoma *in situ* |

Correspondence: *R. Sainsbury, at new address: Huddersfield Royal Infirmary, Huddersfield, HD3 3EA, W. Yorks.
Received 13 March 1988; and in revised form, 14 June 1988.

**Table II** Histology, EGF receptor and oestrogenreceptor status, size and lymph node status of 25 non-ductal breast cancers.

| | *EGFr* | *ER* | *Size (cm)* | *Lymph node status* |
|---|---|---|---|---|
| Lobular $n=14$ | + | − | 3.3 | — |
| | + | − | 2.0 | 2/3 |
| | + | − | 4.5 | — |
| (21% EGFr +ve) | − | + | 3.5 | — |
| | − | + | 2.0 | — |
| | − | + | 5.0 | 1/2 |
| | − | + | 9.0 | 6/7 |
| | − | + | 2.5 | — |
| | − | + | 3.0 | 1/5 |
| | − | − | 1.4 | — |
| | − | − | 8.0 | 1/1 |
| | − | − | 0.7 | — |
| | − | − | 4.0 | 6/6 |
| | − | − | 2.5 | 6/6 |
| Mucoid $n=3$ | + | − | 4.1 | — |
| | − | + | 5.1 | — |
| | − | − | 2.5 | 1/16 |
| Tubular $n=3$ | − | + | 2.4 | — |
| | − | + | 1.8 | 1/5 |
| | − | + | 1.5 | — |
| Cystosarcoma phylloides | + | − | 14.0 | — |
| | − | + | 10.5 | — |
| Clear cell | − | − | 2.5 | — |
| Carcinoid | − | + | 1.7 | 3/3 |
| Fibrosarcoma | − | − | 3.5 | — |
| $n=25$ | 5 EGFr +ve | | 12 ER +ve | |

**Table III** Relationship of EGF receptor and oestrogen receptor for ductal and lobular cancers.

| | *EGFr* Positive | *EGFr* Negative | |
|---|---|---|---|
| *Ductal* | | | |
| ER positive | 10 | 84 | |
| negative | 71 | 74 | |
| | | | 239 |
| $\chi^2=35.7$; $P<0.001$ | | | |
| *Lobular* | | | |
| ER positive | 0 | 6 | |
| negative | 3 | 5 | |
| | | | 14 |
| *Histology* | | | |
| Ductal | 81 | 158 | |
| Lobular | 3 | 11 | |
| | | | 253 |
| $\chi^2=0.44$; $P=0.5$ | | | |

(21%) had EGFr (all ER negative), 6 (43%) were ER positive (and EGFr negative) and the remaining 5 were negative for both receptors. All three tubular lesions were ER positive and EGFr negative. The cystosarcoma phylloides lesions had undergone sarcomatous change – one was EGFr+, ER− and the other ER+, EGFr−.

The previously reported finding of an inverse relationship for the ductal lesions is shown in Table III along with the $\chi^2$ analysis for lobular lesions. Thirty-four percent of these lesions contained EGF receptors.

All the patients in the non-ductal group of tumours with detectable EGFr have experienced recurrence within 2 years of excision.

### Discussion

The association of a worse outlook for patients with cancers containing EGFr has been found for tumours of breast (Sainsbury *et al.*, 1987), bladder (Neal *et al.*, 1985) and stomach (Tahara *et al.*, 1986). Only in the case of the uterus is there a report of a better outcome (Hofman *et al.*, 1984). Although the highest expression of EGFr appears to be in squamous carcinomas there are reports showing that EGFr are found in approximately 35% of breast tumours. Several studies have shown the inverse relationship between EGFr and ER (Sainsbury *et al.*, 1985*b*; Peres *et al.*, 1984; Battaglia *et al.*, 1988).

Increased EGFr expression is associated with enhancement of tumour growth when cell lines are implanted into nude mice (Filmus *et al.*, 1987). This enhanced growth is seen even if nude mice are innoculated with EGFr positive cell lines that do not secrete excess EGF or TGFα suggesting that increased numbers of EGFr are sensitive to endogenous, background, growth factors. Some tumour cell lines do secrete TGFα so there may be a mixture of autocrine and paracrine stimulation. It has been postulated that the stroma in which the epithelial cells sit may be releasing growth factors. T47D cells secrete a PDGF-like substance which may be responsible for the intense schirrous reaction seen round some lesions (Rozengurt *et al.*, 1985).

EGFr expression is related to markers of poor differentiation and there is a correlation with higher Bloom and Richardson scores for ductal carcinomas (Sainsbury *et al.*, 1985*c*).

Overexpression of other oncogenes is also related to poor prognosis – there are recent reports of the *neu* oncogene being overexpressed in tumours which relapsed early (Slamon *et al.*, 1987). There are preliminary data (Wright, unpublished) showing that tumours staining positively for *neu* are associated with early recurrence and death. This finding was independent of EGFr status and the combination of *neu* positivity with EGFr increased the prognostic power.

The histological sub-type of breast cancer allows the pathologist to provide some indication of prognosis. Tubular lesions, along with other tumours with special histological features were found in patients who survived long term whereas tumours with no special histological feature were found in cases which died early. Cribriform tumours were found in 13.4% of long term survivors but in no patient who died in under ten years whilst tumours of no special feature were found in 27.7% of the long term survivors and in 83% of those who died early (Dixon *et al.*, 1985).

Our data, combined with those of Skoog *et al.* (1986), suggest that the histologically high risk tumours are associated with positive EGFr. It appears that EGFr positivity in any histological subtype may be associated with poor prognosis. Furthermore, these EGFr positive lesions are more likely to recur early.

This work was supported by the North of England Cancer Research Campaign.

### References

BATTAGLIA, F., SCAMBIA, G., ROSSI, S. & 8 others (1988). Epidermal growth factor receptor in human breast cancer: Correlation with steroid hormone receptors and axillary lymph node involvement. *Eur. J. Cancer Clin. Oncol.* (in press).

BENSON, E.A. & HOLDAWAY, I.M. (1981). Insulin receptors in human cancer. *Br. J. Cancer*, **44**, 917.

BLOOM, H.J.G. & RICHARDSON, W.W. (1957). Histological grading and prognosis in breast cancer. *Br. J. Cancer*, **13**, 359.

DICKSON, R.B., McMANAWAY, M.E. & LIPPMANN, M.E. (1986). Estrogen-induced factor of breast cancer cells partially replace estrogen to promote tumor growth. *Science*, **232**, 1540.

DIXON, J.M., PAGE, D.L. & ANDERSON, T.J. & 4 others (1985). Long-term survivors after breast cancer. *Br. J. Surg.*, **72**, 445.

DOWNWARD, J., YARDEN, Y., MAYES, E. & 0 others (1984). Close similarity of epidermal growth factor receptopr and v-erb-B oncogene protein sequences. *Nature*, **307**, 521.

FILMUS, J., TRENT, J.M., POLLAK, M.N. & BUICK, R.N. (1987). Epidermal growth factor receptor gene-amplified MDA-468 breast cancer cell line and its non amplified variants. *Molec. Cell. Biol.*, **7**, 251.

FITZPATRICK, S.L., LACHANCE, M.P. & SCHULTZ, G.S. (1984*a*). Characterization of epidermal growth factor receptor and action on human breast cancer cells in culture. *Cancer Res.*, **44**, 3442.

FITZPATRICK, S.L., BRIGHTWELL, J., WITTLIFF, J.L., BARROWS, G.H. & SCHULTZ, G.S. (1984*b*). Epidermal growth factor binding by breast tumour biopsies and relationship to oestrogen receptor and progestin receptor levels. *Cancer Res.*, **44**, 3448.

HOFFMAN, G.E., RAO, C.V., BARROWS, G.H., SCHULTZ, G.S. & SANFILIPPO, J.S. (1984). Binding sites for epidermal growth factor in human uterine tissues and leiomyomas. *J. Clin. Endocrinol. Metab.*, **58**, 880.

HORWITZ, K.B. & McGUIRE, W.L. (1978). Estrogen control of progesterone receptor in human breast cancer. *J. Biol. Chem.*, **253**, 2223.

HOWELL, A., BARNES, D.M., HARLAND, R.N.L. & 0 others (1984). Steroid-hormone receptors and survival after first relapse in breast cancer. *Lancet*, **i**, 588.

JENSON, E.V., DESOMBRE, E.R. & JUNGBLUT, P.W. (1967). Estrogen receptors in hormone responsive tissues and tumours. In *Endogenous Factors Influencing Host-Tumor Balance*, Wissler *et al.* (eds) p. 15. University of Chicago Press: Chicago.

MAYNARD, P.V. & GRIFFITHS, K. (1979). Clinical, pathological and biochemical aspects of the oestrogen receptor in primary human breast cancer. In *Steroid Receptor Assay in Human Breast Tumours: Methodological and Clinical Aspects*. King, R.J.B. (ed) p. 86. Alpha Omega: Cardiff.

McGUIRE, W.L., CHAMNESS, G.C., HORWITZ, K. & ZAVA, D.L. (1978). Hormones and their receptors in breast cancer. In *Receptors and Hormone Action*. O'Malley, B. & Birnbamuer, L. (eds) p. 401. Academic Press: New York.

MURPHAY, L.J., VRHOVSEK, E., SUTHERLAND, R.L. & LAZARUS, L. (1984). Growth hormone binding to cultured human breast cancer cells. *J. Clin. Endocrinol. Metab.*, **58**, 149.

NICHOLSON, S., SAINSBURY, J.R.C., NEEDHAM, G.K., CHAMBERS, P., FARNDON, J.R. & HARRIS, A.L. (1988). Quantitative assays of epidermal growth factor receptor in human breast cancer: Cut off points of clinical relevance. *Int. J. Cancer*, **42**, 36.

NEAL, D.E., MARSH, C., BENNETT, M.K. & 4 others (1985). Epidermal-growth-factor receptors in human bladder cancer: comparison of invasive and superficial tumours. *Lancet*, **i**, 366.

PERES, R., PASCUAL, M., MACIAS, A. & LAGE, A. (1984). Epidermal growth factor receptors in human breast cancer. *Breast Cancer Res. Treat.*, **4**, 189.

ROZENGURT, E., SINNETT-SMITH, J. & TAYLOR-PAPADIMITRIOU, J. (1985). Production of PDGF-like growth factor by breast cancer cell lines. *Int. J. Cancer*, **36**, 247.

SAINSBURY, J.R.C., SHERBET, G.V., FARNDON, J.R. & HARRIS, A.L. (1985*a*). Epidermal growth factors are present on human breast cancers. *Br. J. Surg.*, **72**, 186.

SAINSBURY, J.R.C., FARNDON, J.R., SHERBET, G.V. & HARRIS, A.L. (1985*b*). Epidermal growth factor receptors and oestrogen receptors in human breast cancers. *Lancet*, **i**, 364.

SAINSBURY, J.R.C., MALCOLM, A., APPLETON, D., FARNDON, J.R. & HARRIS, A.L. (1985*c*). Presence of epidermal growth factor receptors as an indicator of poor prognosis in patients with breast cancer. *J. Clin. Pathol.*, **38**, 1225.

SAINSBURY, J.R.C., NEEDHAM, G.K., MALCOLM, A., FARNDON, J.R. & HARRIS, A.L. (1987). Epidermal growth factor receptor status as predictor of early recurrence of and death from breast cancer. *Lancet*, **i**, 1398.

SALOMON, D.S., ZWIEBEL, J.A., BANO, M., LOSCONCZY, I., FEHNEL, P. & KIDWELL, W.R. (1984). Presence of transforming growth factors in human breast cancer cells. *Cancer Res.*, **44**, 4069.

SHIU, R.P. (1979). Prolactin receptors in human breast cancer cells in long term tissue culture. *Cancer Res.*, **39**, 4381.

SKOOG, L., MACIAS, A., AZAVEDO, E., LOMBARDERO, J. & KLINTERBERG, C. (1986). Receptors for EGF and oestradiol and thymidinekinase activity in different histological subgroups of human mammary carcinomas. *Br. J. Cancer*, **54**, 271.

SLAMON, D.J., CLARK, G.M., WONG, S.G., LEVIN, W.J., ULLRICH, A. & McGUIRE, W.L. (1987). Human breast cancer: Correlation of relapse and survival with amplification of the HER-2/neu oncogene. *Science*, **235**, 177.

STEWART, J.F., KING, R.J., SEXTON, S.A. & REUBENS, R. (1980). Oestrogen receptors, sites of metastatic disease and survival in recurrent breast cancer. *Eur. J. Cancer*, **17**, 449.

TAHARA, E., SUMIYOSHI, H., HATA, J. & 5 others (1986). Human epidermal growth-factor in gastric cancer as a biologic marker of high malignancy. *Jpn. J. Cancer*, **77**, 145.